# High Strain Rate Response of In-Situ TiB_2_/7055 Composite by Taylor Impact

**DOI:** 10.3390/ma14020258

**Published:** 2021-01-07

**Authors:** Hengfu Li, Zhenyu Yu, Peng Rong, Yi Wu, Xulong Hui, Fengguo Zhang, Zhe Chen, Haowei Wang

**Affiliations:** 1 State Key Laboratary of Metal Matrix Composites, Shanghai Jiao Tong University, Shanghai 200240, China; lihengfu@sjtu.edu.cn (H.L.); eagle51@sjtu.edu.cn (Y.W.); hwwang@sjtu.edu.cn (H.W.); 2 Chengdu Aircraft Industry (Group) Co. Ltd, Chengdu 610091, China; yzyzy920822@163.com (Z.Y.); rongpeng-love@163.com (P.R.); 3 Aircraft Strength Research Institute of China, Xi’an 710065, China; 13028512013@163.com

**Keywords:** particle reinforced aluminum matrix composite, Taylor impact, flow stress, microstructure evolution, finite element model

## Abstract

The high strain rate deformation behavior and microstructure evolution of in situ TiB_2_ particle reinforced Al-Zn-Mg-Cu composite were investigated by means of Taylor impact. The dynamic tests were performed at three different impact velocities. Under three different velocities, no obvious shear failure occurred in the composite, indicating a good impact resistance. Compared to the quasi-static compression test, the dynamic yield strength increased obviously with the rise of velocity, even more than 1 GPa. The dislocation multiplication, phonon drag effect and ceramic reinforcement increased the flow stress of composite. Fine, equiaxed grain structure developed after impact, resulting from grain fragmentation or dynamic recrystallization. Finite element simulation of Taylor impact was qualitatively in agreement with the experiments, which was useful to elucidate the formation of equiaxed grain structure.

## 1. Introduction

Particulate-reinforced aluminum matrix composites (PRAMCs) have attracted attention due to their high strength, elastic modulus, hardness and good wear resistance compared with aluminum alloys [1,2]. At present, there are two main methods to fabricate PRAMCs: adding particles into the matrix (ex situ) and in situ growth [3,4,5,6]. The in situ method is to select the suitable glume (molecule or aggregate of molecules) and synthesize the reinforcing phase in situ by means of the chemical reaction between matrix and glume at appropriate temperature. In contrast to ex situ synthesized composites, in situ synthesized particulate-reinforced aluminum matrix composites, in particular, have the advantages of fine particle size and strong interface bonding, thus they have prospective uses in aerospace, the automobile industry and precision instruments. Several approaches are used to synthesize in situ TiB_2_/aluminum composites. Among these approaches, the salt-melt reaction technique is simple and economical [3], and it can produce particles with the correct stoichiometry [7]. In certain critical circumstances, the materials will likely undergo high-speed dynamic impacts, such as bird strikes, car collisions, et cetera, thus it is particularly important to study the deformation behavior of composites under high strain rate.

At present, intensive studies have focused on the mechanical behavior [8,9,10,11,12], microstructure evolution [13,14,15,16] and failure behavior [17,18,19] of alloys and composites under high strain rate by means of medium and high strain rate tension, Hopkinson bar, Taylor impact and finite element simulation [20,21,22,23,24]. Acosta et al. [20] developed a reliable method for obtaining the material constitutive parameters. The method was to transform the equivalent plastic strain in Taylor impact into Vickers hardness, and compare it with the results of nano-indentation of AISI 1010. Kumagai [23] assumed that the force on the undeformed volume of the projectile depended on the strain of the deformed volume, and developed the simple equation proposed by Hawkyard to predict the shapes of projectiles after Taylor impact tests more accurately. Chen et al. [12] fabricated 316 L stainless steel by cold metal transfer wire and arc additive manufacturing (CMT-WAAM) process and carried out the impact test. The as-built 316 L dynamically exhibits ~50% higher yield strength and twice the elastic modulus than in the quasi-static condition. Savage [16] studied the texture evolution of WE43 magnesium-rare earth alloy by Taylor impact. The material exhibited homogeneous and isotropic response over the strain rates, and the peak texture components after the impact had their c-axes closely aligned with the impact direction. Zhu et al. [10] found that the dynamic deformation behavior of high-volume fraction (up to 60%) PRAMCs was affected by strain rate hardening and adiabatic heating softening mechanism, which eventually led to a mixture of brittle cracking of reinforcing phase and ductile fracture of matrix alloy in the composite. Zhou et al. [25] studied the microstructure evolution of nano-twinned steel during ballistic impact by nano-indentation, scanning electron microscope (SEM), transmission electron microscope (TEM) and other techniques and they found that high strain rate promoted dislocation multiplication (the activation of dislocation source leading to the increase of dislocation density) and phonon drag effect of dislocation movement (induced by interactions between mobile dislocation and lattice vibration), remarkably increasing the hardness in the severe deformation zone. Obvious recrystallization occurred in the severe deformation zone due to temporarily to the significant temperature rise. In summary, previous studies mainly focused on the verification and modification of constitutive models in the process of high-speed impact. The evolution of microstructure and hard ceramic particles such as TiB_2_ in the deformation process were still inadequate.

The main purpose of this paper is to study the mechanical behavior and microstructural evolution of TiB_2_/7055Al matrix composites under high strain rate by Taylor impact test, and establish the internal relationship between the mechanical properties and microstructure evolution of the composite. The finite element simulation method is introduced to provide insights into the microstructure evolution of the composite.

## 2. Material and Experiment

The material used for this study was 6 wt % TiB_2_ particle reinforced Al-Zn-Mg-Cu composite fabricated by in situ mixed salt method as described in [26]. With an atomic ratio in accordance with Ti/2B, the mixed salts of K_2_TiF_6_ (CAS number: 16919-27-0) and KBF_4_ (CAS number: 14075-53-7) were introduced into a stirred Al melt during the synthesis. The exothermal reactions between the introduced salts and the molten Al are as follows:

$$\{\begin{array}{c} 3K_{2}\mathrm{Ti}F_{6}+13\mathrm{Al}=3\mathrm{Ti}{\mathrm{Al}}_{3}+K_{3}\mathrm{Al}F_{6}+3K\mathrm{Al}F_{4}\\ 2KBF_{4}+3\mathrm{Al}=\mathrm{Al}B_{2}+2K\mathrm{Al}F_{4}\\ \mathrm{Al}B_{2}+\mathrm{Ti}{\mathrm{Al}}_{3}=\mathrm{Ti}B_{2}+4\mathrm{Al} \end{array}$$

**Figure 1:**
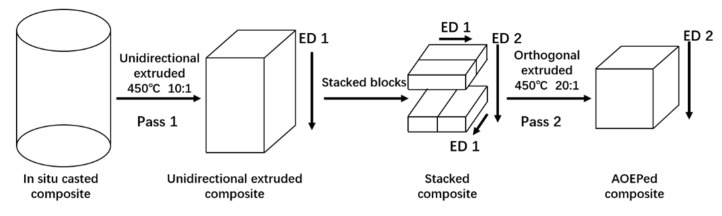
Schematic of accumulative orthogonal extrusion process (AOEP). ED: extrusion direction.

The as-casted composites were processed by a novel two-pass accumulative orthogonal extrusion process (AOEP) [27]. Figure 1 shows the AOEP process. Firstly, the in situ casted composites were unidirectionally extruded at 723 K with an extrusion ratio of 10:1. The extruded composites were then sliced into small blocks from the center of extruded square plates with the longest side parallel to the extrusion direction. Two small blocks were polished and subsequently put together to form a square block. Several square blocks were stacked to form a square column. This stacked square column was orthogonally extruded to a plate at 723 K. After AOEP, the composites were subjected to solid solution at 748 K for 1 h, quenched in water, firstly aged at 383 K for 8 h and secondly aged at 438 K for 3 h.

Quasi-static compression testing was carried out at a strain rate of 1 mm/min. The compression equipment was equipped with a 100 kN load cell sensor (SANS TESTING MACHINE CO., LTD, Shenzhen, China). Six specimens with a diameter of 5 mm and a height of 5 mm were cut from the extruded bars by wire electrical discharging machining and then mechanically grinded by 2500 grit silicon carbide paper.

**Figure 2:**
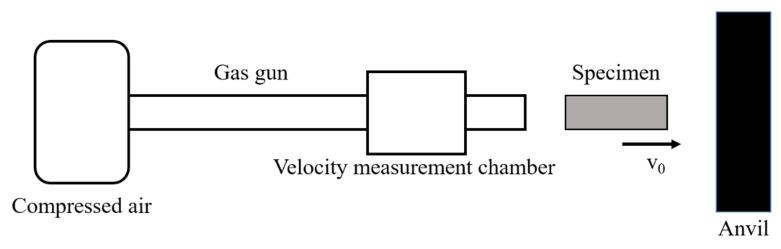
Schematic of a Taylor impact equipment.

The specimens for Taylor impact testing were flat cylindrical projectiles with a diameter of 8 mm and a length of 24 mm (aspect ratio 3:1). The impact velocity was 159.4, 190.4 and 221.7 m/s, measured from the high-speed camera (Photron, Tokyo, Japan), respectively. Figure 2 is the schematic of the commonly used Taylor impact equipment, which can reach a strain rate up to 10^5^ s^−1^, to study the dynamic deformation behavior and microstructure evolution at high strain rate.

## 3. Result and Discussion

### 3.1. Microstructure of the AOEPed Samples

**Figure 3:**
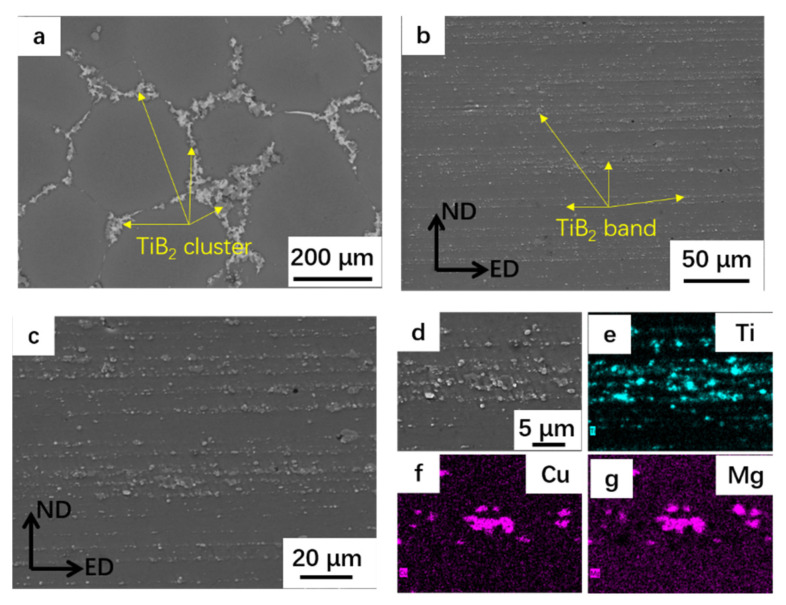
Scanning electron microscope (SEM) micrographs of composite:(**a**) as-cast composites; (**b**) AOEPed composites; (**c**,**d**) are the magnification images; and (**e**–**g**) are the corresponding energy dispersive X-ray spectrometer (EDX) mappings of Ti, Cu and Mg in (**d**). ED: extrusion direction; ND: normal direction.

In as-cast composites, the particles tended to be pushed by the solid-liquid interface front and agglomerate at the grain boundary, as shown in Figure 3a [26]. Figure 3b,c shows the SEM images after two-pass of extrusion. The results indicated that the agglomeration of the particles was well dispersed. The particles were mainly aligned along the extrusion direction (ED), forming particle bands. Figure 3d–g shows that some ternary phases consisting of Al, Cu and Mg were also distributed along the extrusion direction around the TiB_2_ particles.

**Figure 4:**
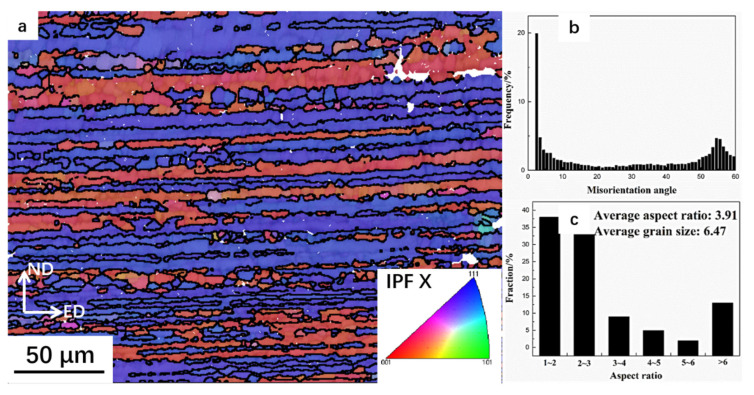
(**a**) Electron backscattered diffraction (EBSD) maps of the specimens after AOEP colored by inverse pole figure (IPF) with axis parallel to the extrusion direction; (**b**) the misorientation angle distribution and (**c**) aspect ratio and average grain size.

Figure 4a–c respectively shows the EBSD (electron backscattered diffraction) maps colored by IPF (inverse pole figure) with axis parallel to the extrusion direction, the relevant misorientations and aspect ratios of grains in the AOEP samples. Coarse columnar grains lay parallel to ED and fine grains co-existed in the AOEPed composite, respectively. After AOEP, low angle grain boundary dominated, as shown in Figure 4b.

### 3.2. Quasi-Static Compression Test

**Figure 5:**
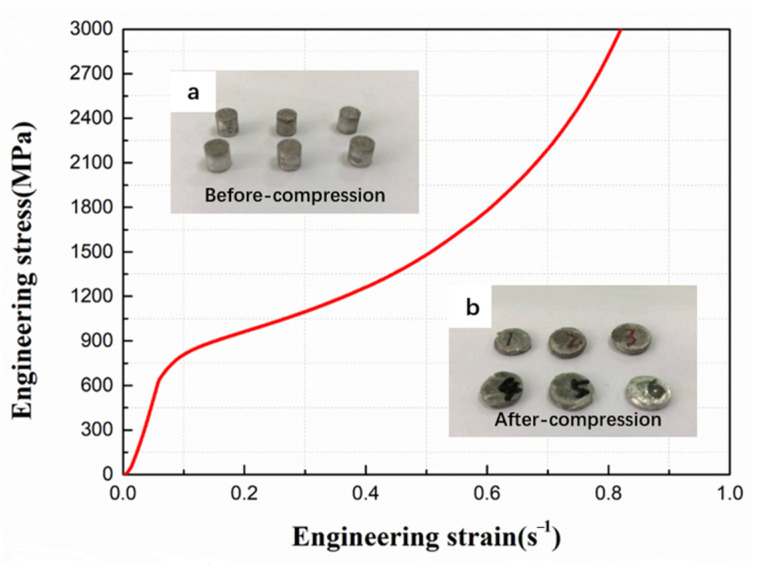
Representative stress–strain curves of AOEP specimens at a strain rate of 1 × 10^−4^ s^−1^. The inset figure is the specimens before and after quasi-static compression for (**a**) and (**b**), respectively.

Six specimens were tested for quasi-static compression. Figure 5 shows one representative quasi-static compressive stress–strain curve of the composite. The stress–strain curve was marked by three distinctive regions. In the plastic region, the material exhibited high and uniform work hardening rate, the flow stress increased uniformly with the increase of strain. The stress–strain curves exhibited no obvious yield plateau. We therefore took $\sigma_{0.2}$ as the yield strength of the composite, yielding a static yield strength of 673 MPa. When the load exceeded a critical value, the flow stress increased rapidly with strain due to the intense opposition between two sides of the specimen, as shown in the specimens after compression in Figure 5b.

### 3.3. Taylor Impact Test

**Figure 6:**
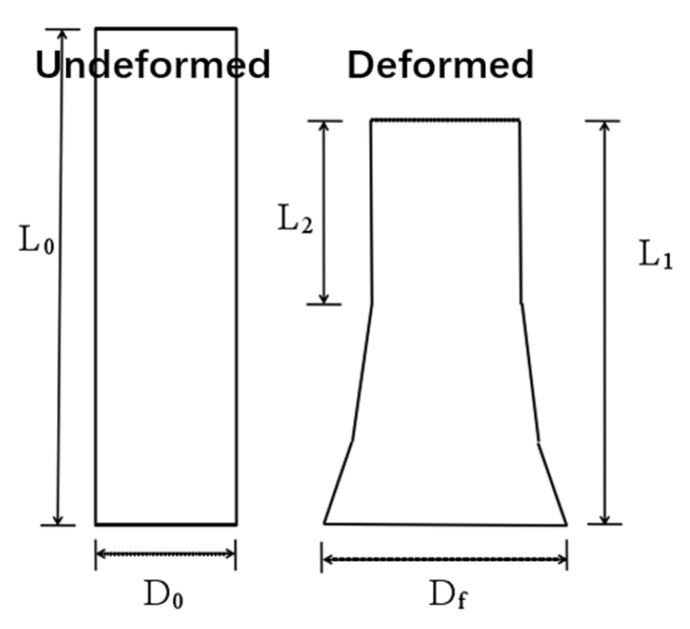
Schematic of the Taylor impact specimen before and after impact.

Taylor testing can be used to calculate the yield strength of materials at high strain rate (up to 10^5^ s ^−1^). Figure 6 is the schematic of the Taylor impact specimen before and after impact, from which the dynamic yield strength of the material can be estimated [28], with Equation (1):

(1) $$\sigma_{s}=\frac{\rho_{\mathrm{mat}}V^{2}\left(L_{0}-L_{2}\right)}{2\left(L_{0}-L_{1}\right)\ln\frac{L_{0}}{L_{2}}}$$

where σ_s_ is the dynamic yield strength; $\rho_{\mathrm{mat}}$ is the material density; *V* is the impact velocity; *L*_0_, *L*_1_ and *L*_2_ are initial length, final length and unstrained length, respectively.

The average strain and strain-rate can be calculated using Equations (2) and (3), respectively [29]:

(2) $$\varepsilon =\frac{L_{0}-L_{1}}{L_{0}-L_{2}}$$

(3) $$\dot{\varepsilon }=\frac{V}{2\left(L_{0}-L_{2}\right)}$$

Wilkins et al. proposed a simplified model to estimate the dynamic yield strength of materials [30]:

(4) $$\ln\frac{L_{1}}{L_{0}}=-\frac{\rho_{\mathrm{mat}}V^{2}}{2\sigma_{s}}$$

Other authors introduced a parameter $\frac{h}{L_{0}}$ to further modify the model, where the value could be regarded as a constant value to be 0.12:

(5) $$\frac{L_{1}}{L_{0}}=\left(1-\frac{h}{L_{0}}\right)e^{\left(-\frac{\rho_{\mathrm{mat}}V^{2}}{2\sigma_{s}}\right)}+\frac{h}{L_{0}}$$

**Figure 7:**
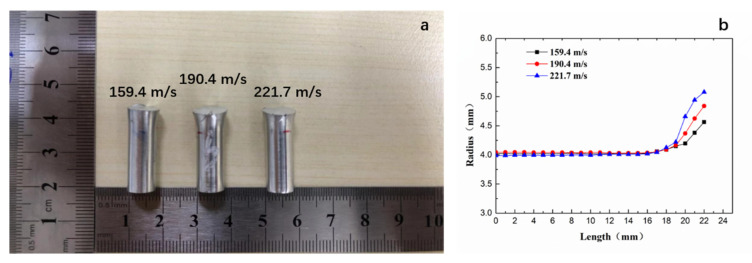
(**a**,**b**) the shape and the silhouettes of the specimens at different impact velocities.

Figure 7a,b shows the macroscopic morphology and silhouettes of the composite at different impact velocities, respectively. After deformation, the shape of the specimens typically assumed mushroom shapes, without noticeable shear fracture, indicating good impact resistance. With the increase of impact velocity, the specimen height *L*_1_ decreased and the final diameter *D*_f_ increased.

**Figure 8:**
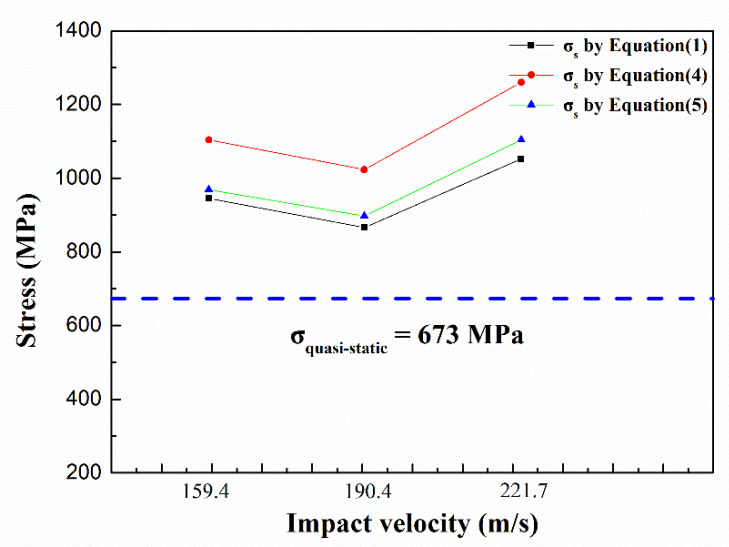
Dynamic yield stress calculated by three equations. The column in black was calculated by Equation (1), the column in red was calculated by Equation (4) and the column in green was calculated by Equation (5). The blue dotted line represented the quasi-static yield strength.

The experimental dynamic yield stresses were calculated using the contour of the specimens in Table 1. Figure 8 shows the dynamic yield stress after different impact velocities by Equations (1), (4) and (5), respectively.

It can be seen from Figure 7 that the strain rate had a remarkable influence on flow stress. The dynamic yield strength (>800 MPa) of the material was significantly higher than the quasi-static yield strength (673 MPa). An abnormal case in which the yield strength at 190.4 m/s was higher than the yield strength at 159.4 m/s occurred. Further investigations are needed to test and verify the strain rate sensitivity and the mechanism. After all, the sampling of the velocities was inadequate to clarify the dependence of strength on the strain rate in the present study. However, the yield strength of the material was obviously enhanced at high speed, even more than 1 GPa.

The traditional constitutive model was used to understand the increasing yield stress of the composite.

First, the flow stress can be decomposed as [31]:

(6) $$\sigma =\sigma_{0}+\sigma_{f}$$

where σ_0_ is the friction stress due to the solid solution and σ_f_ is the stress induced by forest dislocations. Forest dislocations refer to the entangled dislocations which hinder the movement of the mobile dislocations.

Additionally, σ_f_ can be described by [32]:

(7) $$\sigma_{f}=\alpha Gb\sqrt{\rho_{t}}$$

where α is a constant value related to the interaction strength between dislocations, *G* is the shear modulus, *b* is the Burgers vector and $\rho_{t}$ is the total dislocation density, containing mobile dislocations and forest dislocation.

During the deformation, the applied strain rate should be equal to the strain rate inside, which is linked to the mobile dislocation density and the velocity of the mobile dislocation, as in Equation (8):

(8) $$\dot{\varepsilon }=\rho_{m}bv_{d}$$

where $\rho_{m}$ is the mobile dislocation density and $v_{d}$ is the velocity of mobile dislocation.

In order to match the strain rate inside the material with the applied strain rate, the dislocation density and the dislocation velocity will change significantly. At relatively low strain rate, the mobile dislocation density will increase with the applied strain rate, giving rise to the total dislocation density. This will make $\sigma_{f}$ increase according to Equation (7), resulting in the increase of flow stress. When the strain rate is higher, the density of the mobile dislocations will be saturated and their speeds are increased accordingly, making the interactions among dislocations, particles, grain boundaries and precipitates more frequent. The flow stress will rise as a result. When the strain rate exceeds 10^3^ s^−1^, the velocity of the mobile dislocation is sufficiently high, phonon drag effect induced by the interaction between lattice vibration and the mobile dislocations plays an important role in the increase of yield strength, which is neglected at low strain rate [33]. All the factors mentioned above account for the increase in the dynamic yield strength. Due to the high stacking fault energy of the Al alloy, deformation by twinning was unlikely and not observed in the experimental results.

The effect of TiB_2_ particle on the flow stress cannot be ignored at all strain rates. Due to the introduction of particles into the composites, the following two factors probably lead to the increase of strength. Firstly, the misfit strain induced by the incoherency of atomic arrangement at the interface increased the density of dislocations; secondly, at high strain rates, particles could significantly hinder the movement of dislocations, resulting in the increase of dislocation resistance.

### 3.4. Grain Structure Variation during Impacting

**Figure 9:**
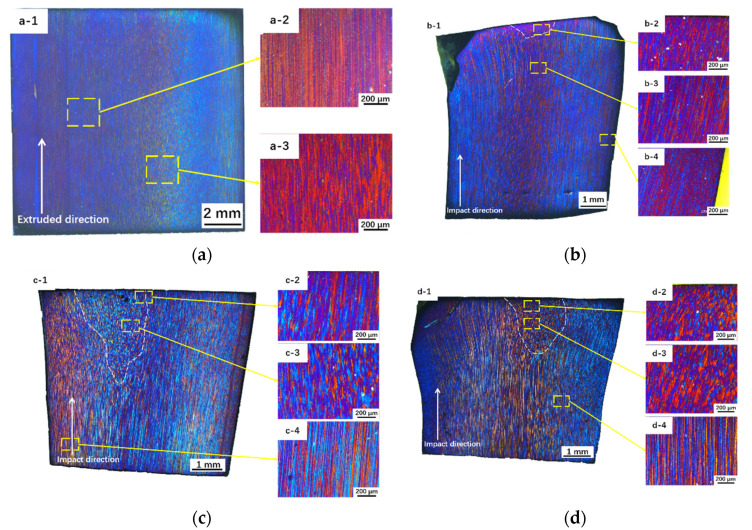
Polarized metallographic structure of the specimens after impact at different velocities: (**a**) AOEP, (**a-2**) and (**a-3**) are enlarged pictures of (**a-1**), which show the typical elongation grains along the extrusion direction; (**b**) *v* = 159.4 m/s; (**c**) *v* = 190.4 m/s; and (**d**) *v* = 221.7 m/s; After impact, the specimen mainly contains two regions: severe deformation region and small deformation zone. (**b-2**,**b-3**,**b-4**) are the enlarged pictures of the severe deformation region, the boundary and the small deformation zone in (**b-1**), respectively. (**c**) and (**d**) are the same as (**b**).

Figure 9 shows the polarization microscopy of grain structure of the material after different impact velocities. Figure 9(a-1,b-1,c-1,d-1) were composed of several polarized images to show the entire microstructure distribution of the specimen, respectively. Figure 9a shows the microstructure before deformation. Corresponding to the SEM and EBSD results, the projectile showed typical extrusion grain structure. When the specimen was impacted at *v* = 159.4 m/s, the grain did not seem to change seriously. At the end of the specimen, the grains were distorted, evidently in contrast to the initial specimens. When the velocity reached 190.4 m/s, the grain structure changed significantly. It mainly contained two areas: extrusion texture grains at the bottom and sides of the specimen and equiaxed grains at the end and the center of the specimen. The closer the area to the head and the center, the smaller the grain size was. Two reasons that may account for the formation of the equiaxed grains: (i) Dynamic impact results in an increase in the material energy, which increases the driving force of recrystallization (especially rotational dynamic recrystallization because of such a short time interval) [34]. (ii) The local area of the composite underwent an extremely high deformation in such a short time, so the primary grains were sheared and broken to adapt to macro-deformation.

### 3.5. Particle Variation during Impacting

**Figure 10:**
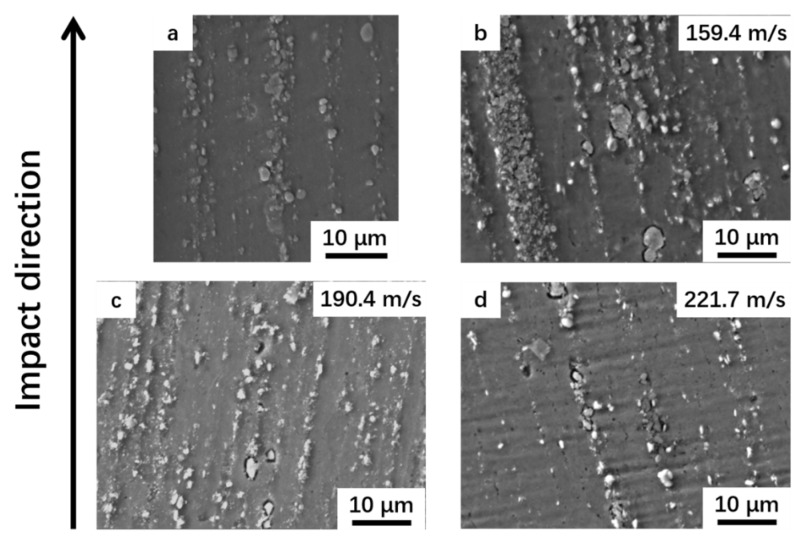
SEM micrographs of specimens (**a**) before impact; and after impact at different velocities: (**b**) *v* = 159.4 m/s; (**c**) *v* = 190.4 m/s and (**d**) *v* = 221.7 m/s.

Figure 10 shows the particle distribution of the specimens before and after impact. It is observed that the dynamic impact has little effect on particle distribution. The distribution band of particles deviated from the direction of extrusion and impact. In contrast to precipitates such as the  θ ′ phase which will be decomposed by the dislocation cutting and redissolving in the Al-Cu alloy [35], and brittle fracture of ex situ particles [10], the in situ TiB_2_ particles in this study did not show any obvious brittle fracturing under dynamic loading, due to the high modulus of the ceramics particles and small size thanks to the in situ synthetization. Meanwhile, the TiB_2_ particle had a good coherency with the Al matrix and a clean interface, preventing cracks from debonding at the interface.

### 3.6. Taylor Impact Test Finite Element Model

The finite element simulations of the Taylor impact process at different impact speeds were carried out by ABAQUS/Explicit software. The constitutive model was a Johnson–Cook model (JC model) of aluminum alloy [36]. The JC model was a phenomenological constitutive model which considered the strength as a function of strain, strain rate and temperature.

The Johnson–Cook equation is as follows:

(9) $$\sigma =\left(A+B\varepsilon^{n}\right)\left(1+C\ln\left(1+\frac{\dot{\varepsilon }}{\dot{\varepsilon_{0}}}\right)\right)\left(1-{\left(\frac{T-T_{\mathrm{room}}}{T_{\mathrm{melt}}-T_{\mathrm{room}}}\right)}^{m}\right)$$

where B and n are strain hardening coefficients, C is strain rate sensitivity and m is the thermal softening coefficient.

At room temperature, $T=T_{\mathrm{room}}$, the equation can be simplified as:

(10) $$\sigma =\left(A+B\varepsilon^{n}\right)\left(1+C\ln\left(1+\frac{\dot{\varepsilon }}{\dot{\varepsilon_{0}}}\right)\right)$$

The main parameters used in the finite element models are shown in Table 2. *E* is the elastic modulus and ν is the Poisson’s ratio. Parameters of the JC model were calculated by fitting the compression stress–strain curve of the composite.

**Figure 11:**
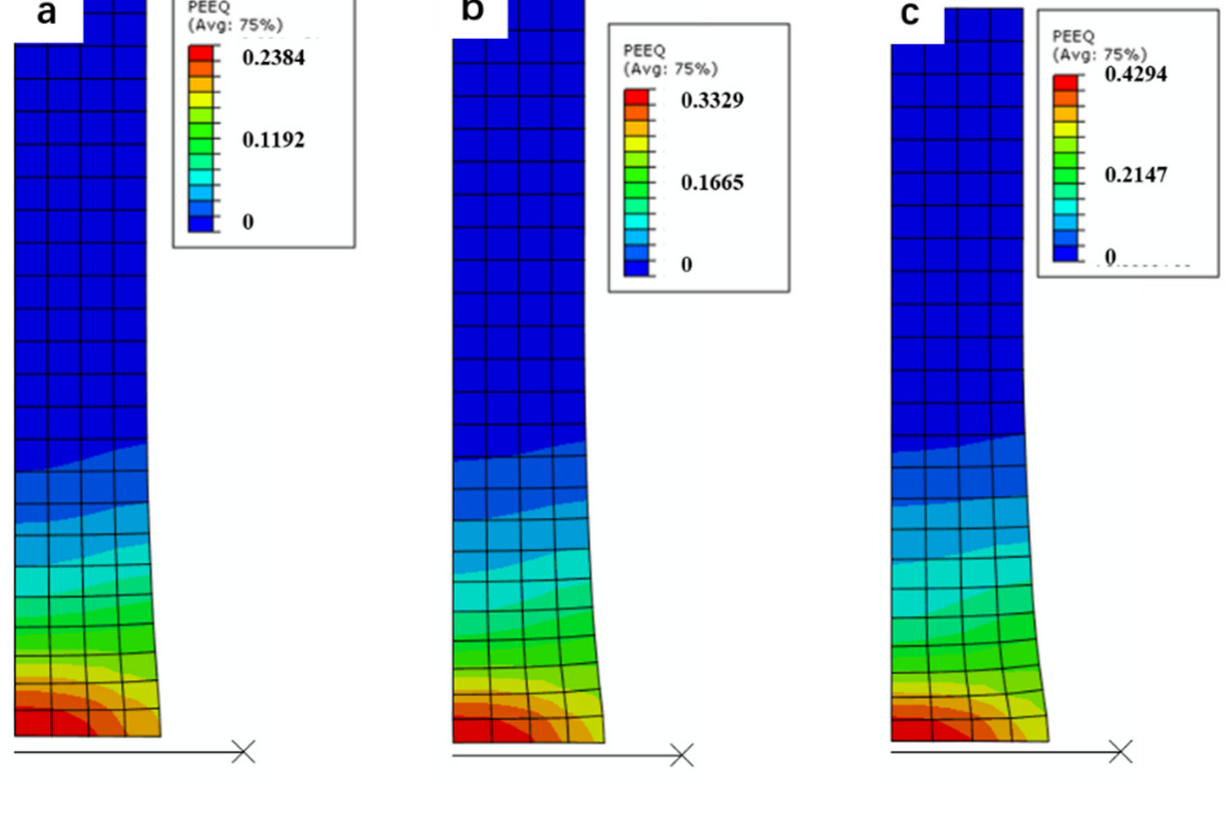
Effective plastic strain contour plot at different velocities: (**a**) *v* = 159.4 m/s; (**b**) *v* = 190.4 m/s and (**c**) *v* = 221.7 m/s.

The simulation results of Taylor impact were qualitatively in agreement with the experimental results. As shown in Figure 11. The results of finite element simulation show that the deformation of the specimen is mainly concentrated in the head of the projectile, and the most severe deformation occurred at the center of the head. However, no obvious plastic deformation occurred in the unstrained region. The strain distribution in simulation was consistent with the microstructure evolution shown in Figure 9, in which the microstructure evolution (formation of equiaxed grain) mainly occurred at the head of the specimens and the rest remained unchanged. The large strain at the head may account for the microstructure evolution. Meanwhile, as shown in Table 3, the height of the specimens after impact decreased with the increase of the impact velocity, which showed a similar trend as the experiment results in Table 1. The above two aspects confirmed the reliability of our simulation results. However, the results of finite element simulation showed a discrepancy with the experiment quantitatively. The height of deformed specimen in simulation is slightly lower than that of the experiment, which may be caused by two factors: (i) The contact between the anvil and the specimen was not ideally flat, resulting in deviation axisymmetric deformation, and hence, overestimation of the measured specimen length; (ii) JC model was relatively simple, and the effect of particles on material strength enhancement was not considered in the model, so the simulation results were lower than the real value. Future work will focus on matching the results between simulation and experiment by changing the parameters in the constitutive model, so as to obtain the real constitutive model parameters of the composite.

## 4. Conclusions

In this study, in situ TiB_2_ particle reinforced aluminum matrix composites were prepared, and the aggregation of particles in the composites was dispersed by a two-pass AOEP process. The quasi-static compression tests and Taylor impact tests were carried out to study the mechanical behavior and microstructure evolution of the composite. Based on ABAQUS/Explicit software, the JC equation was used as the constitutive model to simulate the impact process. The main conclusions were as follows:

- The dynamic yield strength of the composite was significantly higher than that of the quasi-static compression, and there was no shear fracture in the composite. This was mainly due to the phonon drag effect at high strain rate, more frequent interaction among dislocations, particles, grain boundaries, et cetera, and the increased hindrance to dislocation motion.
- After impact, the grain structure in the head of the specimen changed obviously, from the original typical extruded grain structure (mixed structure of large grains and small grains) to the nearly equiaxed grain structure, due to the severe deformation at the head, resulting in grain breakage or rotational dynamic recrystallization during high strain rate deformation.
- SEM images show that the particle distribution of the composites did not change significantly after impact, still mainly forming particle bands. The direction of the band changed. There was no brittle fracture of TiB_2_ particles and debonding of the interface with the matrix, showing better impact resistance of the composite offered by the TiB_2_ particles.
- The finite element results show that the simulation results by JC model were in agreement with the experimental results. During Taylor impact, the deformation was mainly concentrated in the head, especially in the center. With the increase of impact velocity, the degree of local plastic deformation increased significantly (from 0.238 to 0.429), which led to the microstructural transformation.

Future research will be focused on: the strain rate sensitivity of the composite; further analysis on fine, equiaxed grain structure after impact by means of TKD (transmission Kikuchi diffraction) and TEM (transmission electron microscopy) equipment; and refinement of the constitutive model for composites.

## Figures and Tables

**Table 1:** The size of the Taylor impact specimens at different velocities.

*L*_1_/mm	*L*_2_/mm	*D*_f_/mm	*V* (m/s)
23.2	16.79	9.11	159.4
22.78	16.06	9.56	190.4
22.66	15.51	10.18	221.7

**Table 2:** Parameter used in Taylor impact finite element model.

ρ_mat_(kg/m^3^)	*E* (GPa)	ν	Parameters of JC Model				
			*A*	*B*	*n*	*m*	*T*
2700	68	0.33	657	542	0.76	0.5	933

**Table 3:** The main results from Taylor impact finite element model.

*V* (m/s)	Height (mm)	Maximum of Effective Plastic Strain
0	24	-
159.4	22.217	0.238
190.4	21.845	0.333
221.7	21.484	0.429

## Data Availability

Data available in a publicly accessible repository.
